# Proteases of Wood Rot Fungi with Emphasis on the Genus *Pleurotus*

**DOI:** 10.1155/2015/290161

**Published:** 2015-06-09

**Authors:** Fabíola Dorneles Inácio, Roselene Oliveira Ferreira, Caroline Aparecida Vaz de Araujo, Tatiane Brugnari, Rafael Castoldi, Rosane Marina Peralta, Cristina Giatti Marques de Souza

**Affiliations:** ^1^Laboratory of Biochemistry of Microorganisms, Department of Biochemistry, State University of Maringá, Avenue Colombo 5790, 87015-900 Maringá, PR, Brazil; ^2^Federal Institute of Paraná, Campus Jacarezinho, Avenue Doutor Tito s/n, Jardim Panorama, 86400-000 Jacarezinho, PR, Brazil

## Abstract

Proteases are present in all living organisms and they play an important role in physiological conditions. Cell growth and death, blood clotting, and immune defense are all examples of the importance of proteases in maintaining homeostasis. There is growing interest in proteases due to their use for industrial purposes. The search for proteases with specific characteristics is designed to reduce production costs and to find suitable properties for certain industrial sectors, as well as good producing organisms. Ninety percent of commercialized proteases are obtained from microbial sources and proteases from macromycetes have recently gained prominence in the search for new enzymes with specific characteristics. The production of proteases from saprophytic basidiomycetes has led to the identification of various classes of proteases. The genus *Pleurotus* has been extensively studied because of its ligninolytic enzymes. The characteristics of this genus are easy cultivation techniques, high yield, low nutrient requirements, and excellent adaptation. There are few studies in the literature about proteases of *Pleurotus* spp. This review gathers together information about proteases, especially those derived from basidiomycetes, and aims at stimulating further research about fungal proteases because of their physiological importance and their application in various industries such as biotechnology and medicine.

## 1. Introduction

Enzymes are increasingly required in the commercial and industrial fields. For this reason, there is an intense search for new enzymes with particular properties that are desirable for certain commercial applications [1]. There are a limited number of known enzymes that are used commercially and consequently, the enzymes that are available are not used in large quantities. Approximately 75% of industrial enzymes are hydrolases, and the enzymes which degrade proteins account for 65% of the enzymes that are marketed worldwide [2].

Proteases catalyze hydrolytic reactions, in which protein molecules are degraded into peptides and amino acids. Their properties are very diverse because the group is large and complex [3]. The study of proteases is of note in enzymology because of its biotechnological relevance. Proteases are a special group of enzymes because of their importance in the metabolism of organisms, their biochemical functions in metabolic pathways and cellular signaling, the importance of protease inhibitors, and their use in fine chemicals and the pharmaceutical industry [4].

Most of the proteases used industrially are microbial and especially bacterial origin and these are preferred for their desired characteristics in biotechnology and their lower cost. Proteases which are of plant and animal origin, except for some specific uses, do not meet industrial demand. The industrial production of microbial proteases is favored due to the fact that they have a short generation time, because of the ease of genetically manipulating microorganisms, and because of the diversity of species available in nature, many of which are still unexplored [2, 3].

Because of their potential therapeutic use, genes from protease bacteria, fungi, and viruses have been cloned and sequenced in order to increase the production of enzymes by recombinant DNA technology, to study the role of enzymes in pathogenicity and to cause changes in the properties of proteases to improve their commercial usage. In industries, proteases contribute to the development of processes and products with high added value. As biological catalysts, they offer advantages in relation to the use of chemical catalysts for numerous reasons, such as high catalytic activity, high specificity, and their availability in economically viable quantities [5]. However, the cost of production of proteases is the greatest barrier to their industrial application. Consequently, researches have been conducted to find low cost proteases useful in commercial and industrial sectors [6].

Bacteria produce the majority of proteases of microbial origin. The genus* Bacillus* produces proteases which are mainly neutral and alkaline [7]. However, the proteases of fungal origin,* Aspergillus* [8] and* Penicillium* [5], as well as being widely studied, appear in greater variety. A species can produce neutral, acidic, or alkaline proteases, as is the case of* Aspergillus oryzae* [7]. Proteases from basidiomycetes have unique properties and deserve further study. Although scientific research regarding the structural and functional characteristics of proteases from basidiomycetes started more than 30 years ago, the diversity and complexity of action of these enzymes has resulted in recent studies of xylotrophic basidiomycetes as a new source of proteases [9].

The* Pleurotus* species are highly appreciated in cooking for their refined flavour and they have also been investigated because they contain bioactive, antitumor, anti-inflammatory, hypocholesterolemic, antiviral, antibiotic, antioxidant, antidiabetic, immunomodulatory, antitumor, antihyperlipidemic, and hepatoprotective compounds, among others [3, 10–12].

The genus* Pleurotus* is also known for its ability to degrade lignin through the production of ligninolytic enzymes, particularly laccase [11, 13]. Several studies have been performed with laccase of* Pleurotus* spp. [14, 15], linking the metabolism of ligninolytic enzymes with the presence of proteases [16]. Besides laccase, the production of numerous hydrolytic enzymes by such organisms has also been reported [17], and interesting studies of the proteases produced by* Pleurotus* spp. have been described, resulting in the need for further research on these properties of this genus. The aim of this review was to gather information on proteolytic enzymes, including their most relevant and current industrial applications, as well as to gather the characteristics of proteases obtained from basidiomycetes, especially from the genus* Pleurotus*.

## 2. Uses and Applications of Proteases

### 2.1. In the Detergent Industry

The use of proteases as a detergent dates back to 1914, when the “Burnus” brand of detergent was produced, which contained sodium carbonate and pancreatic extract [7]. Proteases can be separated into two major groups according to their ability to cleave N- or C-terminal peptide bonds (exopeptidases) or internal peptide bonds (endopeptidases), the latter being those which are most important industrially. They are also classified according to their optimum pH for activity (acid, neutral, or alkaline) and substrate specificity (collagenase, elastase, keratinase, etc.). Based on their mechanism of action and the functional groups in the active site, proteases can be classified into four main groups: serine, cysteine, aspartate, and metalloprotease [9].

There are several industries that benefit from the catalytic properties of proteases, such as pharmaceutical, chemical, food processing, detergents, leather processing, and others. Their use in bioremediation processes has also been explored. Their properties, such as substrate specificity, optimum temperature and pH for activity, stability, and catalytic mechanism, differ greatly because this group is quite diverse [9, 18].

The proteases used in detergents need to have stability in wide ranges of pH and at high temperatures, as well as compatibility with oxidizing agents. Interest in proteases that are active in a wide temperature range has been increasing because garments made from synthetic fibers are sensitive to high temperatures [19]. However, although bacterial proteases are commonly used in detergents, the high cost of the cell separation process, that is, obtaining cell-free enzyme preparations, limits their use. In this context, enzymes of fungal origin have advantages because they are mainly extracellular. Furthermore, the use of proteases as a basis for detergents is preferable to conventional synthetic products because they have greater cleaning capacity, improved performance at low wash temperatures, and reduce pollution because they are natural. Thus, there is always a demand for enzymes with improved efficiency that can improve the performance of detergents containing enzymes [7].

### 2.2. In the Pharmaceutical and Food Industries

Many proteases are related to the processes of infection caused by viruses, bacteria, and fungi, which are central to the interaction with the host cell. Proteolytic reactions are finely regulated and the variety of mechanisms involve high substrate specificity, ATP-directed protein degradation, restricted access to the active site, activation cascade, and selective and highly specific protein modification, as can be seen in the activation of zymogenic forms of enzymes by limited proteolysis [18, 20].

The involvement of proteases in the mechanisms that cause diseases has caused them to become a target for developing therapeutic agents against diseases such as AIDS, cancer, Chagas disease, hepatitis, malaria and candidiasis, as well as inflammatory, immune, respiratory, cardiovascular, and neurodegenerative disorders [7, 18].

Natural inhibitors play a role in the regulation of the proteolytic activity in cells; hence knowledge about the interaction of proteases with their substrates and their specificity is an essential tool for the development of synthetic inhibitors that can be used to control diseases in which proteases are involved [21]. There is an emerging market for enzyme inhibitors in countries like India, China, Japan, South Korea, Taiwan, Canada, Australia, and New Zealand [22]. Studies of the protein structure of peptidases through X-ray diffraction have made the development of proteolytic inhibitors possible by molecular modeling [21]. The first successful examples of protease inhibitors were the inhibitors of the aspartic protease of HIV-1, which were developed by the modeling technique. The peptidase of HIV cleaves the polyproteins of the virus into structural proteins, which are essential for the production of mature, infectious viral particles [23].

The accumulation of fibrin in the blood can lead to thrombosis, which can cause heart attacks and other cardiovascular diseases [24]. Many products currently used in thrombolytic clinical therapy have undesirable side effects, such as intestinal bleeding in oral treatments, low specificity to fibrin, and relatively high costs [25–27]. Consequently, the growing interest in obtaining fibrinolytic proteases at a reduced cost and with the appropriate medical characteristics has led researchers to intensify their studies and, in recent decades, a number of fibrinolytic enzymes were isolated and characterized. Enzymes with fibrinolytic capacity have been obtained from snake venom, insects, marine animals, algae, fermented products, and microorganisms that are safe for humans and animals (food grade) [28–39].

Recently, the fibrinolytic activity of proteases produced by microorganisms has attracted greater medical and commercial interest. Microorganisms are important sources of thrombolytic agents, though few of them have GRAS status (“generally recognized as safe”, i.e., totally safe for humans and animals, and the products obtained from them). Some species of* Bacillus* produce enzymes with thrombolytic activity, such as nattokinase (NK) from* Bacillus natto*, subtilisin DFE, and subtilisin DFE DJ-4 from* Bacillus amyloliquefaciens* [39]. Likewise,* Streptococcus hemolyticus* produces a streptokinase with thrombolytic action [26, 40]. In recent years, the search has intensified for microorganisms producing proteases with fibrinolytic activity and which are “food grade,” with the potential for exploiting them as functional additives in food and drugs to prevent or treat thrombosis and other related organic disorders [39].

Other therapeutic agents include proteases that are used in the correction of deficient digestive enzymes. Elastase is used in the treatment of wounds, burns and abscesses [41, 42]. Proteases also play important roles in the production of animal feed, cleaning contact lenses, silver recovery from photographic films and X-ray and in the treatment of domestic and industrial sewage [19, 43].

One of the most important industries in which proteases play an essential role is the food industry. They act as agents for modifying the functional properties of proteins, particularly in the processing of cheese (milk clotting, by the hydrolysis of a specific binding in casein), in obtaining protein hydrolyzates, improving the flavor of some foods and also in baking [19]. Proteases from* Aspergillus oryzae* are used to modify the gluten of wheat flour by facilitating handling and increasing the volume of bread dough. Proteases have been used since ancient times to prepare sauce and other derivatives from soy because this grain has high, good quality protein content. The proteolytic modification of soy proteins helps to improve their functional properties. These enzymes are also used in the synthesis of the artificial sweetener aspartame (through synthesis reactions produced by the thermolysin of* B. thermoprotyolyticus*) and in the maturation (softening) of meat, particularly beef, through the alkaline elastase action of* Bacillus*. The microorganisms most commonly used for the production of proteases in the food industry are from the genus* Bacillus* [7, 18, 26, 40].

The requirements for proteases to act as industrial catalysts vary considerably. The enzymes to be used in the production of detergents and in the food industries need be produced in large quantities and should be efficient without further processing (*in natura*). The proteases already used in the pharmaceutical industries (such as medicines) are produced in small amounts but require extensive purification procedures [18].

## 3. Proteases of Fungal Origin

The cost of production of proteases is the biggest obstacle to their industrial application. Consequently, the development of new processes to increase the yield of proteases with respect to industrial production, concomitantly with reduced production costs, is highly advantageous from the commercial point of view. Increased productivity has been achieved by selecting hyper-productive strains or by improving the culture media [7]. The global market for industrial enzymes reached about US $4.5 billion in 2012, with a projection of US $7.1 billion for 2018. Research on enzymes has revealed their use in different sectors and their catalytic properties have stimulated their use in industrial production and processes. Market growth has been positively influenced by new products and their advantages over traditional industrial methods [22, 44].

Although the species of microorganisms that are used for industrial production are few in number, 90% of commercialized proteases are obtained from microbial sources. These are preferred to proteases from plants and animals due to their various characteristics, which are more suitable for biotechnological applications, such as activity within a broad range of temperature and pH, thermal stability, and high catalytic activity [18, 41, 45].

Biodiversity is an invaluable resource for biotechnological innovation and it promotes the search for new strains of microorganisms to be used for specific industrial purposes. Because the use of proteases, especially those of the alkaline variety, is expected to rise over the coming decades, the production of microbial proteases represents a good alternative for the development of new methods in order to improve the production of these enzymes, as well as decreasing their price [7, 19]. The increased demand for proteases with specific properties has led biotechnologists to explore new sources of proteases.

Most fungal proteases have neutral to slightly acidic characteristics [19]. Xerophilic fungi often contain proteases of low molecular weight (26 to 50 kDa) [24]. The study of fungal proteases has increased in recent decades, but knowledge about proteases from basidiomycetes is still limited [9]. In 2009, approximately 60% of the enzymes commercialized originated from fungi and only five originate macrofungi (three laccases, one peroxidase, and one phytase) [11].

A few years ago, the proteases produced from micromycetes were predominant in studies regarding the search for new bioactives with economic and medicinal benefits [24].* Aspergillus* is considered to be the best producer of proteases [8]. In the food industry* A. oryzae* and* A. sojae* are noteworthy for their ability to eliminate bitterness [19].* Penicillium *and* Rhizopus* are also considered to produce proteases [5, 46] and the proteases from macromycetes recently gained prominence in the search for new enzymes with specific characteristics. Proteases produced from basidiomycetes such as* Agaricus bisporus*,* Armillariella mellea*,* Flammulina velutipes*,* Grifola frondosa*,* Pleurotus ostreatus*,* Pleurotus eryngii*,* Phanerochaete chrysosporium*,* Schizophyllum commune*, and others have been reported [47–50].

There are vast majority of microorganisms that exist in nature have not yet been studied. Thus, the search for new natural molecules with interesting physiological effects, and the need to understand the mechanisms of production and regulation of expression of these bioactives, has resulted in the fact that the cultivation conditions that have already been defined and used successfully for ascomycetes such as* Aspergillus* sp. and* Penicillium* sp. have now been extended to include basidiomycetes in the search for secondary bioactives and metabolites such as enzymes, antibiotics, and organic acids [46].

## 4. Proteases from Basidiomycetes

Basidiomycetes are fungi important for biological communities because they are excellent at degrading wood. Some genera have been used as food for centuries and they have enormous commercial importance. They are also producers of a group of commonly studied extracellular enzymes (xylanases, cellulases, and ligninolytic enzymes) [51]. Proteases play important roles in the physiology of fungi, acting in processes such as germination and sporulation. These enzymes seem to have a close relationship with the lifestyle of saprophytic fungi, as observed in* Pleurotus citrinopileatus* [43].

It has been found that* P. pulmonarius*, which usually grows on dead timber, secretes subtilisin but does not produce trypsin.* P. ostreatus*, which grows in living hosts, secretes extracellular trypsin throughout its development. The presence of living tissues as hosts may be related to the expression of trypsin-type proteases [24]. Because most of the nitrogen in timber is in the form of proteins, proteases play a very important role in the metabolism of the proteins in the fungi of white rot in wood and it has been observed that depletion of nitrogen in the medium stimulates the secretion of proteases by fungi [3, 24, 52–57].

The mycelial secretion of proteases by saprophytic basidiomycetes has led to the identification of various classes of proteases: subtilases were found in* Pleurotus ostreatus* [58],* Phanerochaete chrysosporium* [59],* Serpula lacrymans* [52],* Schizophyllum commune* [47], and* Coprinus *sp. [60]. Metalloproteinase was reported by Mchenry et al. [61] in* Chondrostereum purpureum* and in* Hypsizygus marmoreus* [62]. The mycelial secretion of aspartate proteases was reported in* P. chrysosporium* [49, 59],* Amanita muscaria* [63], and* Irpex lacteus* [64].

Although they are recognized for their nutritional value and the extraction of bioactive compounds of basidiome and mycelia, mushrooms still possess much unexplored information in relation to some of the enzymes that they produce, such as proteases [6]. Proteases extracted from mushrooms have been purified and characterized [48]. The role of proteases in the regulation of the formation of basidiome in* Hypsizygus marmoreus* was described by Terashita et al. [62] and their regulatory role regarding ligninolytic activity in* P. chrysosporium* and* P. ostreatus*, under nutritional limitation, was highlighted by Dass et al. [59] and Palmieri et al. [16], respectively.* Phanerochaete chrysosporium* has produced an acid protease in solid medium with wood, under ligninolytic conditions. This enzyme showed an isoelectric point that was higher than that of most acid proteases (5.6) and it has been characterized as a glycoprotein aspartate protease [49].

From a selection of 27 strains of basidiomycetes that produce proteases [3],* Lentinula edodes* stood out with the largest halo of proteolytic activity using the method of selection on plates containing casein. In this study, the genus* Pleurotus* ranked second in the production of protease. The authors attributed this proteolytic activity to ability of the fungus to grow on substrates with low nitrogen availability [47]. However, Zorn et al. [65] consider that the existence of nitrogen seems to stimulate the production of proteases by fungi. Media containing soybean, casein, gelatin, corn, and yeast are commonly used to produce protease. Other sources, such as starch, lactose, and barley are also used, but it is known that high concentrations of carbohydrates inhibit the production of enzymes [19]. The purification of a fibrinolytic protease from* Cordyceps militaris* showed characteristics of a 52 kDa subtilisin, which was higher than other fungal proteases. The enzyme rapidly degraded the *α* and *γ* chains, but it took longer to degrade the *β* chains of the fibrin, which was a pattern quite different from the action of proteases derived from snake venom [27]. The fibrinolytic protease activity of basidiomycetes has been recently demonstrated by several authors. Kim et al. [66] purified and characterized a metalloprotease from the mycelium of* Perenniporia fraxinea* with fibrinolytic activity. The cloning, purification, and characterization of proteases from* Pleurotus ostreatus* with similar characteristics were performed by Yin et al. [2], Shen et al. [67], and Joh et al. [68].

Although several studies have performed the purification and characterization of proteases from mycelium, basidiome, or culture filtrate, many aspects of the production of these enzymes have yet to be explored. The process of producing basidiome is laborious and time-consuming; it requires large volumes of substrate, space, and qualified labor and these factors hinder research in the laboratory. Cultivations which are performed in the vegetative phase are more viable for research because they can be kept in the laboratory, performed on a small and medium scale, and important parameters such as temperature, humidity, and agitation can be controlled [18].

Microbial proteases can be produced in various ways and studies have shown that, depending on the culture conditions, different forms of the same protease can be expressed [19]. Most of the enzymes produced in industry are produced by submerged fermentation [8]. The use of liquid cultures facilitates the purification of bioactives such as enzymes and polysaccharides [3]. The submerged culture of* P. ostreatus* in wheat gluten resulted in the secretion of proteases that noticeably increased the overall solubility of the medium [13].

Submerged media with complex sources provide higher yields of protease compared to simple media, such as casein or gelatin [19]. However, using submerged culture requires greater resources, specific strains, and very controlled conditions, which does not occur in solid state fermentation, which therefore offers advantages in terms of environmental and economic aspects [8]. The solid cultivation of mushrooms and mycelium in order to obtain bioactives and enzymes remains a very viable alternative; waste from agriculture, forestry, or municipal waste are used for the production of enzymes of industrial interest. The combination of different solid substrates sometimes appears to increase the production of protease by fungi [19].

Due to the similarity of the natural habitat of basidiomycetes, these organisms have excelled in the production of enzymes in solid cultures. Solid state fermentation in tomato pulp yielded good colonization and protease production on a large scale using* P. ostreatus* [8]. Furthermore, proteases have been obtained by the solid state fermentation of soybean and wheat bran fibers [69]. The literature includes standardized techniques for the high yield recovery of proteases, as well as immobilization methods and different protocols for proteolytic assays and the purification of proteases [19].

## 5. Proteases from* Pleurotus* spp

The genus* Pleurotus* is the second main group of cultivated edible mushrooms in the world, comprising more than 40 species [51]. In descending order of worldwide production, the seven most produced edible mushrooms are* Agaricus bisporus*,* Pleurotus* spp.,* Lentinula edodes*,* Auricularia *spp.,* Volvariella volvacea*,* Flammulina velutipes*, and* Tremella fuciformis* [11]. However, species of the genus* Pleurotus* present advantages when compared with others mushrooms. For example, they can be cultivated in different substrates and temperatures. They are also rich in essential amino acids and vitamins [70].* Pleurotus* can be grown artificially without major problems and it grows in a disorderly manner in tropical and subtropical regions [51]. This genus is a part of ligninolytic organisms and several studies have reported the ligninolytic capacity of its species [65]. Several bioactive compounds have been extracted from crude extracts, mycelia, and basidiome of* Pleurotus* spp. for study, such as polysaccharides, hemicelluloses, peptides, glycoproteins, lipids, hydrolytic enzymes, and others [12].

Extracts of the basidiome and mycelia of* Pleurotus* spp. have been used as medicines and as nutritional supplements for human health. Several studies have reported its nutritional, immunomodulatory, antioxidant, antitumor, and hypoglycemic properties, among others.* P. ostreatus* has been effective in alleviating the effects of hepatotoxicity in rats and it protects the liver, heart, and brain against oxidative stress [51].

In recent years, examples of the main genera of cultured basidiomycetes have been studied for their positive therapeutic effects. Hepatoprotective effect was observed for* P. pulmonarius*,* P. ferulae*, and* P. tuber-regium*, which were also active against human cancer cells [71]. Moreover, species of the genus have been used in the processes of bioremediation, delignification, and disinfection of effluent [17].

**Figure 1:**
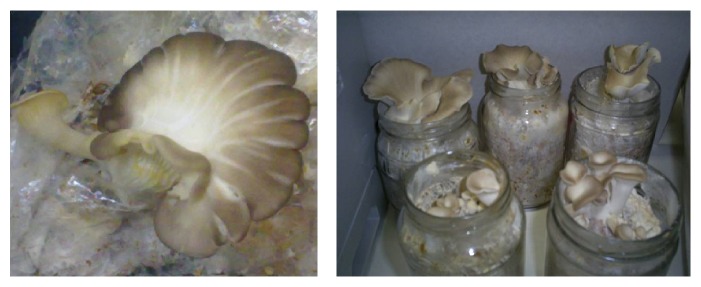
*Pleurotus pulmonarius*.

Different strains of* Pleurotus* spp. (Figure 1) exhibit specific behaviors, which vary depending on the conditions where they are cultivated, including environmental factors such as types of substrates and supplementation. A study of three strains of* P. eryngii* using sawdust and rice straw as a substrate for cultivation showed significant differences between the strains regarding growth rates, number of days for the first harvest, biological efficiency, and other parameters [72].* P. eryngii* is considered as one of the best species of the genus due to its consistency and because it has a longer lifetime than all the other species of* Pleurotus*. While most of the fungi in the Agaricales order show steady growth in tree trunks,* P. eryngii* grows well in subtropical pastures and grows excellently during cold periods [72].

The artificial cultivation of* P. eryngii* on farms using automatic devices and sawdust has been performed successfully in Korea. The species has also been effective in lowering the levels of blood glucose, the inhibition of tumor cells, and antioxidant activity [73].* P. citrinopileatus* contains polysaccharides that have antihyperglycemic and antitumor effects [43]. Medicinal properties have also been observed in* P. tuber-regium*, which is also edible and grows well in tropical and subtropical regions. Several of its bioactive substances have been identified, such as glycoproteins, polysaccharides, and phytochemicals with pharmacological action [74].

Basidiome of* P. pulmonarius* has shown antitumor, antioxidant, and anti-inflammatory properties, suggesting the therapeutic effects of its metabolites in the treatment against some diseases, such as cancer [75].* P. sajor-caju* and* P. ostreatus* have also been investigated for their antioxidant capacity and both species share a similar amino acid profile. However, despite similarities with the properties of* Pleurotus *spp., there are many studies of* P. ostreatus* at the expense of other species of the genus [51]. As stated, there is still little knowledge about the proteases derived from mushrooms, mainly the* Pleurotus* genus [11].

Studies of the* P. ostreatus*,* P. eryngii*,* P. citrinopileatus*, and* P. chrysosporium* species have showed that the* Pleurotus* genus is a producer of proteases that seem to participate in the complex ligninolytic mechanism, degrading the laccase enzyme at certain stages of fungal growth [16, 43, 50, 59, 73].

*P. pulmonarius* has important antimicrobial, anti-inflammatory, antioxidant, and antitumor properties; however, there is still little material in the literature regarding its production of proteases and their characterization. Nevertheless, it is known that the proteases secreted by this species do not appear to participate in the regulation of peroxidases, as has been reported for* P. ostreatus* proteases [16, 24, 75, 76]. In addition, no degradation of ligninolytic enzymes was observed when they came into contact with proteases from* Phanerochaete chrysosporium* [49].

In a comparison of six species of basidiomycetes,* P. eryngii* excelled in the production of protease. Pleurerin, the protease extracted from fruiting bodies of* P. eryngii* with anti-HIV-1 action, has presented the characteristics of an aspartic protease due to its N-terminal sequence, which is different from other aspartic fungal proteases [50]. Most proteases of the genus* Pleurotus* feature the characteristics of alkaline subtilases. There are six families of serine proteases, which are based on their amino acid sequence [58]. A study of proteases from 43 species of basidiomycetes showed a predominance of serine protease [9]. An alkaline protease was found in the basidiome of* P. citrinopileatus* and it showed a similarity in amino acid sequence with* Agaricus bisporus*,* Epichloë typhina*, and* Penicillium oxalicum* fungi [43]. Aspartic proteases are divided into 16 groups and are rarer in* Pleurotus* [2].

The fibrinolytic proteases of* Pleurotus* spp. have received attention in recent years because those searching for new proteases with fibrinolytic capacity are interested in nontoxic and edible fungi [77]. A monomeric protease with fibrinolytic activity was purified 29.3-fold from the basidiome of* P. eryngii* produced in corn cob. The protease in question showed a high capacity for degrading fibrin and demonstrated a possible application as a thrombolytic agent. The hydrolysis of *α* and *β* chains of fibrinogen occurred in less than 10 min. The enzyme showed characteristics of a serine protease similar to subtilisin, as has been reported for most proteases from the genus [73].

In a study by Liu et al. [77] the fibrinolytic and fibrinogenolytic enzyme from* P. pulmonarius* grown in submerged state were efficient in degrading the *α* (3 min) and *β* (45 min) chains of fibrinogen, followed by *γ* after 10 h incubation. The enzyme was purified 147-fold and presented good stability at human body temperature, which enables it to be used as an alternative in thrombolytic treatments, including oral applications, because it is an edible fungus. Apart from fibrin degradation, the enzyme was also able to act as a plasminogen activator, which is not common in the literature. A fibrinolytic metalloproteinase purified from mycelia of* P. ostreatus* showed a high similarity with the fibrinolytic proteases from the basidiome of the same fungus, which suggests the need for studies related to therapeutic treatments for thrombosis from the mycelium, which can be obtained more quickly and easily than the fruiting bodies [67].

Hemolytic proteases have been reported less frequently in the genus* Pleurotus*. The hemolysin of the basidiome of* P. nebrodensis* showed apoptosis-inducing activity and antiproliferative cancer cells and also anti-HIV-1 activity, interfering in some way in the permeability of the cell membranes and preventing virus infection [78]. Activity against cancer cells has also been verified for proteases of* P. ostreatus*. Hemolysin extracted from* P. eryngii* was effective against leukemia cells [79] and showed antimicrobial effect for* Bacillus* sp. [80]. However, these enzymes are not stable at temperatures higher than 40°C, which hinders their possible application as a medicament in the form of food because they would be made inactive by cooking or when passing through the intestinal tract [80, 81].

Keratinases are a class of proteases that have received attention in the past. They consist of proteases that are capable of degrading substrates that are rich in insoluble keratins, such as wool, hair, and nail. Because of this, keratinases are used in environmental and technological processes [82]. However, regarding proteases in general, there is little existing research on keratinases of basidiomycetes and there are few reports about keratinases produced by the genus* Pleurotus* spp.

The secretome of* P. sapidus* has shown protease production and ligninolytic enzymes, but some points remained unidentified, indicating that new enzymes with potential biotechnological applications should be studied and identified in the species [65]. The secretome of* P. ostreatus* has shown the presence of proteases with a potential role in the regulation of other extracellular enzymes [13]. Metalloproteases are enzymes that are finely associated with physiological processes and they have been explored in studies of bacteria and mammals; however, there have been very few studies of the metalloproteases of basidiomycetes [68].* Pleurotus* spp. has been greatly cultivated for research related to medicine and also for the consumption of its fruiting bodies, which have agreeable flavors [24].

Although the process of the emergence of the fruiting body of basidiomycetes is still not fully known, the expression of a metalloprotease in the early stages of the formation of a basidiome of* P. ostreatus* has been verified, although the enzyme was not expressed in the mycelial stage or in the formation of spores [68]. The increase of proteases in mature hyphae has also been noted in mycelia of* P. pulmonarius*, linking the time of the formation of the basidiome with the presence of such enzymes [70]. Furthermore, the addition of inhibitors of metalloproteinases has prevented the normal process of the formation of fruiting bodies of* Hypsizygus marmoreus* [62]. Vanillic acid has been reported as a good inducer of proteases of* Pleurotus ostreatus* [58]. In just four days of cultivation in solid state fermentation in residues in tomato,* P. ostreatus* produced high levels of protease, surpassing fungi which are considered to be the best producers of this enzyme, such as* Aspergillus* [8].

The most studied species of the genus is* P. ostreatus*. Proteases of* P. ostreatus* with the capacity to coagulate of milk have been purified [83] and despite the classical techniques of production of proteases, recombinant enzymes have been produced in order to find better yields and new specialties [2, 19, 67, 68].

Recent studies of DNA sequence and proteins have come together with the aim of studying enzymatic structures and mechanisms. As already mentioned, due to the vast diversity of proteases, further knowledge of molecular structures in 3D, active sites and mechanisms of catalysis, and enzyme inhibition are increasingly necessary [18]. Based on the N-terminal sequences of proteases of* P. ostreatus*, a primer was developed in order to clone and amplify a DNA sequence that showed homologous regions with a hypothetical protease of* Neurospora crassa* and another of* Phanerochaete chrysosporium*—the first basidiomycete with a completely sequenced genome [58].

**Figure 2:**
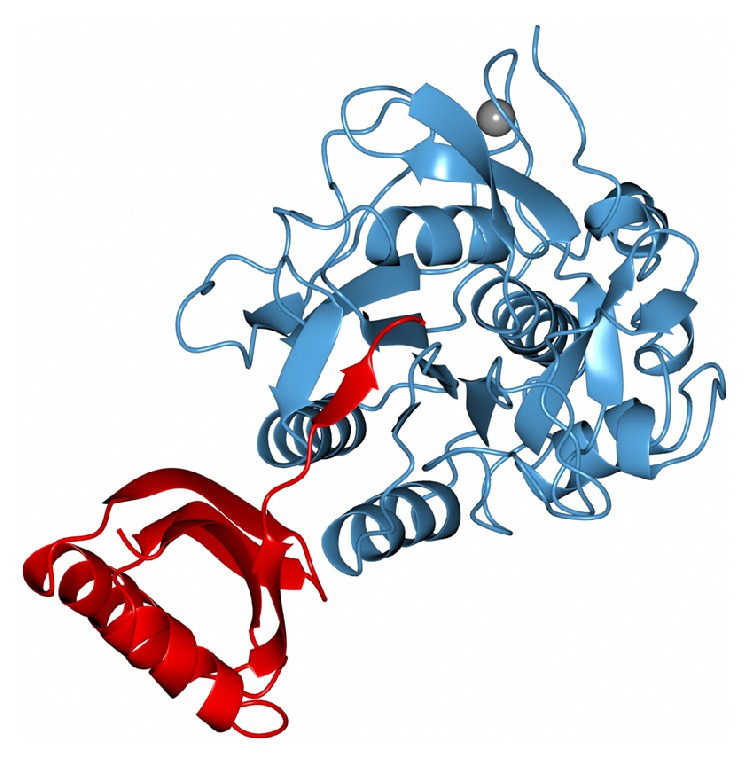
Ribbon model of subtilisin BPN (blue) from* Bacillus amyloliquefaciens* in complex with serine protease inhibitor POIA1 (red) and calcium ion (grey sphere). Figure from pdbid: 1V5I.

A recent study compared the genomes of 33 basidiomycetes and resulted in the idea that the division of fungi into white rot fungi and brown rot fungi in wood cannot be sustained because of the existence of DNA sequences shared between the two groups of fungi that attributes complexity to the mechanisms of degradation of cellulose, hemicellulose, and lignin by basidiomycetes [89]. The three-dimensional structures of proteases and their inhibitors provide rich information about the mechanisms involved in catalysis, and they suggest processes for enzyme inhibition that are still unknown. Using X-ray diffraction, the three-dimensional structure of a serine protease inhibitor of* P. ostreatus* complexed with subtilisin is shown in Figure 2 [84].  Table 1 shows some characteristics of proteases* Pleurotus* spp.

## 6. Conclusions

There is still much progress to be made in the study of proteases of* Pleurotus* spp. and there is still much to be discovered regarding the genome, proteome, and metabolome of the genus. Several proteases of* Pleurotus* spp. have shown unique characteristics, which require further research.* Pleurotus ostreatus* is one of the few edible mushrooms produced on an industrial scale. Most of the research to be found in the literature concerns artificially cultivated basidiomycetes. It is known that there is a high demand in industry for proteolytic enzymes with appropriate specificity and stability to temperature, pH, metal ions, and so forth. However, it is common to find that studies of proteases of basidiomycetes recommend that further, more detailed, studies are required to reveal the mechanisms and physiological effects of proteases. Thus, studies of new proteases of the genus* Pleurotus*, especially wild species, are an area of biotechnology that needs to be explored.

## Figures and Tables

**Table 1:** Characteristics of proteases *Pleurotus* spp.

Species	Molecular weight (kDa)	Optimum pH	Optimum temperature	Kind of protease	References
*P. ostreatus *	43				[2]
*P. ostreatus *var.* florida *	38.7	7.5	37°C	Serine proteinase	[13]
*P. citrinopileatus *	28	10	50°C	Serine proteinase	[43]
*P. eryngii *	11.5	5	45°C	Aspartic protease	[50]
*P. ostreatus *	32	6.5	35°C	Metalloprotease	[67]
*P. eryngii *	14	5	30–40°C	Serine proteinase	[73]
*P. ostreatus *	18.2	7.4	40°C	Metalloprotease	[77]
*P. nebrodensis *	27				[78]
*P. eryngii *	17		37°C		[80]
*P. ostreatus *				Serine proteinase/metalloprotease	[83]
*P. ostreatus *	22	6.7			[84]
*P. ostreatus *	75	7.8		Serine protease	[85]
*P. ostreatus *	30/19/42.5	7.4/5.6		Serine protease/metalloprotease	[86]
*P. sajor-caju *	14.5/86			Metalloprotease	[87]
*P. ostreatus *	97/48.5	5.5–6.5		Cysteine protease	[88]
